# The Impact of Different Factors on Repeat Visits to Selected International Tourism Enterprises: Case Study From Czechia

**DOI:** 10.3389/fpsyg.2022.881319

**Published:** 2022-05-03

**Authors:** Patrik Kajzar

**Affiliations:** Department of Tourism and Leisure Activities, School of Business Administration in Karviná, Silesian University in Opava, Karvina, Czechia

**Keywords:** consumer behavior, factors, service, environment, offer, Czechia, international tourism enterprises

## Abstract

The aim of this article is to identify the influence of the different factors on the repeat visits to selected international tourism enterprises based on the responses of customers in Czechia. The selected factors were divided into three main groups: service, environment, and offer, and each of these groups consists of other different factors. Primary data were analyzed using SPSS software. Statistical hypotheses were formed, reflecting the relationship between the difference in customer responses and selected quality factors, which can affect repeated visits to selected tourism enterprises. Most customers of repeat visits to selected tourism enterprises are affected by professional behavior, staff empathetic approach, offer knowledge and ability to provide as much information as possible, cleanliness and tidiness, overall atmosphere of the establishment, lighting and thermal comfort, air cleanliness, and plenty of space, taste and quality of food and ingredients, and last but not least also corresponding ratio of price/quality, good experience and attractive price level. Studying consumer behavior is important because it helps marketers understand what influences consumers’ buying decisions not only in the tourism area in the Czechia but can fill in the market gap and identify the new products and services that are needed.

## Introduction

The article focuses on current issues, which is useful for analyzing consumer buying behavior and for detecting factors that influence tourism demand. This article becomes even more important in the period of the COVID-19 pandemic. In this respect, it could bring us new significant information on the example of a case study from Czechia.

The COVID-19 pandemic and the restrictions imposed in response have had a massive impact on the hospitality sector worldwide. The bar and restaurant industry has been severely affected by regulations and restrictions, such as the shutdown policy over weeks, issued in many countries (Neise et al., 2021). Therefore, in addition to consumer buying behavior, we must also take into account the COVID-19 pandemic.

Consumer ideology follows the basic definition of the term “ideology,” which is the body of ideas reflecting social needs and aspirations of an individual, a group, a class or culture (Dmitrovic and Vida, 2010, p. 149).

According to Upadhyay et al. (2017), consumer buying behavior is one of the most important areas of study in marketing science. Some of the researchers (Kivela et al., 2000; Kotler and Keller, 2009; Fetscherin, 2014; Holmqvist and Renaud, 2015; Dung et al., 2020) have elucidated their works based on either theory or survey.

According to Kajzar (2021, p. 55–56), travel and tourism are an extremely complex activity for several reasons. First, it relates to goods and tangible products (souvenirs, gifts, food, etc.) as well as intangible services (sightseeing tours, package tour, cultural performances, etc.). Second, the tourism product consists of a multitude of these goods and services put together, and in turn, there exist a multitude of options to choose from within each of the tangible goods and intangible service categories. The tourism services sector is characterized by a wide variety of stakeholders representing services related to catering, transport, accommodation, tourist attractions, etc.

The aim of this article is to identify the influence of the different factors on the repeat visits to selected international tourism enterprises based on the responses of the customers in Czechia.

In the survey, the main factors were divided into three main groups, service, environment and offer, and each of these groups consists of other selected factors. In the first group, we can find factors such as professional behavior, language skills, empathetic approach of staff, offer knowledge, and ability to provide as much information as possible. The level of service and human resources are essential to the success of any business, and even more so in the tourism industry.

The second group is connected to the environment. The environment refers to tangible and intangible factors and conditions that surround selected tourism enterprises. Factors such as facility availability and location, parking, attractive interior design, cleanliness, and tidiness, plenty of space, music production, overall atmosphere of the establishment, the fashionability of the establishment visited, and lighting and thermal comfort, air cleanliness play an important role to retain existing customers and also in attracting new ones.

Factors such as attractive novelties in offer, seasonal offer, taste of food, quality of food and ingredients, the way the food is served, wide range of food and beverages, home cooking, good experience, corresponding ratio of price/quality, etc. belongs to the factors for repeat visits to selected international tourism enterprises in Czechia.

To be better prepared to face these challenges, especially during the COVID-19 pandemic, restaurant owners and managers need to know the factors that influence the choice of restaurants (Janyan, 2018, p. 4).

## Theoretical Background of Consumer Behavior

The history of consumer behavior research is closely intertwined with the history of marketing thought, so each marketing era has had an effect on consumer behavior research (Chrysochou, 2017). Solomon et al. (2002) wrote that consumer behavior is the study of the process involved when individuals or groups select, purchase, use, or dispose of products, services, ideas, or experiences to satisfy needs and desire.

Kotler and Keller (2009) presented a model in which consumer behavior is portrayed to result from the cultural, social, and personal characteristics of consumers, as well as consumer psychology. On the other hand, Renner et al. (2012) developed a comprehensive survey measuring eating motivation that includes 15 dimensions: liking, habits, need/hunger, health, convenience, pleasure, traditional eating, natural concerns, sociability, price, visual appeal, weight control, affect regulation, social norms, and social image. Another model like TPB (Theory of planned behavior) authors Huamin and Xuejing (2019, p. 163) used to explain the behavior and the intention of the behavior. The results of their research demonstrated that 5 factors, including subjective norm, behavior control perception, attitude, activity, destination image, value of tourists, and other variables, have significant influence on leisure tourism intention.

Before the COVID-19 pandemic, dining-out motivations included hunger, social image, health, hedonic value, atmospheric, subjective well-being, celebration, socialization, convenience, natural concerns, traditional eating, price, affect regulation, take-away, and habits (Dedeoglu and Bogan, 2021). The COVID-19 pandemic has substantially affected the restaurant sector, especially businesses whose revenues come mainly from face-to-face service. The global drop in restaurant reservations and on-site consumption in March reached 100% compared to the same period in 2019 in different countries (Hakim et al., 2021). Perhaps the biggest changes have happened in the takeaway market over the last 5–10 years or so, though, has been the rise of home delivery services. These companies promise to connect the consumer (i.e., the home diner) with a range of takeaway options (Spence et al., 2021, p. 2).

According to Kim and Lee (2020), it is important to understand how the perceived threat of COVID-19 affects various behaviors, including the preference for restaurants. They predict that consumers who perceive the threat of COVID-19 as high will prefer private dining restaurants or private tables in a restaurant (Alan et al., 2017). In order to understand how different components, including economic challenges, contribute toward psychological distress. It was developed a scale to measure COVID-19 phobia, which refers to an overwhelming and debilitating fear or anxiety about COVID-19. Some of the most frequently identified reasons behind panic buying include the perception of scarcity and an increased demand for commodities (Seih et al., 2021, p. 2). Which is also important to consider when identifying consumer behavior.

Chi et al. (2022) draw on the Protection Motivation Theory (PMT) to propose and develop a conceptual framework and support their arguments. PMT has been conceptualized as an individual’s response to a fear appeal. During the COVID-19 pandemic, PMT has been applied in communications with the aim of promoting better health outcomes, and PMT also attracted the general attention of behavioral, social, and environmental psychologists. PMT focuses on threats and perceived efficacy; it is based on the premise that people will take action if they perceive a serious threat. Fear of contracting the COVID-19 virus and spreading to loved ones can deter one from making the decision to eat out or travel.

In many parts of the world, restaurants have been forced to close in unprecedented numbers during the various COVID-19 pandemic lockdowns that have paralyzed the hospitality industry globally. This highly challenging operating environment has led to a rapid expansion in the number of high-end restaurants offering for example take-away food, or home-delivery meal kits (Spence et al., 2021, p. 1). Consumers experience products or services, and then the process does not end; it continues. When it comes to restaurants, which constitute an important field in the hospitality sector, determinants of the intention to revisit intention are accepted as perceived quality and satisfaction with the emotions of consumption of the service cape, customer satisfaction and switching barriers customer satisfaction feeling emotion food quality, service quality, atmosphere, price, etc. with customer satisfaction (Alan et al., 2017).

Tourism companies around the world are required to adopt advanced marketing strategies and techniques. Tourism digitization will become a challenge not only for marketing tools that have substantial benefits and influences in different settings and domains (Mathew and Soliman, 2020). As most research on tourism consumer behavior assumes thoroughly planned decisions, the habituated aspects of tourist decision making and its implications for tourism marketing are in urgent need of research (Cohen et al., 2013).

According to Hakim et al. (2021), it is assumed that consumers’ intention to visit restaurants during COVID-19 would be predicted by a set of marketing-oriented stimuli (price, perceived safety, and brand), politically oriented stimuli (social trust, politics, and culture), and perceptions (risk perception) and characteristics (age, employment status). In country-specific factors, such as economic situation, the severity of the pandemic, and national culture, are all parameters affecting the general public’s behavior. Regarding the voluntary and mandated distancing measures taken to regulate physical social interaction between individuals (Atalay and Solmazer, 2021).

## Data and Methods of Research

Within the project related to institutional support “Trends and innovations in tourism in the Czech Republic 2020–2022,” the author has focused, among other things, on the prediction and estimation of the development of the tourism trend in the area of selected tourism enterprises, providing services in Czechia, and also on customers, their preferences, and shopping decision making that has been analyzed within the trends found. This project follows institutional support for “Touristic trends in the Moravian Silesian region (MSR) in 2017–2019” (Kajzar et al., 2020). Another upcoming study is devoted to the issue of trends and innovation from the perspective of selected tourism companies in the Czechia. According to Novotný and Duspiva (2014), they were precisely defined, which determined that the respondent had to be a resident of Czechia and the age of the respondent had to be 15 years and older. Once the criteria were established, a sample of the number of respondents was collected according to the formula for unknown composition of respondents as reported by Kozel (2006).


n⁢(z⁢2*p*q)/Δ⁢2

where n is the minimum number of respondents;

where p, q are the percentage of respondents who are knowledgeable or inclined to choose to option one (p) and ignorant or leaning toward option two (q),

if we do not know these numbers exactly, we have to create the product of the maximum p × q maximum,

i.e., 50% × 50%; where Δ is the maximum allowable error;

where z is the critical value of the normalized normal distribution in the chosen region.

Significance level.

To calculate the minimum sample size, the 95% confidence level (selected quantiles) of the normalized normal distribution can be found for the construction of the 95% confidence interval value 1.96 Hindls et al. (2007) and the margin of error was 5%.

Based on these data, the following result was calculated:


n≥n⁢(1,962*0,5*0,5)/0,052

n≥384

The result shows that in order for the research to be representative according to the chosen criteria, 384 or more participants are required respondents.

The survey was conducted randomly, anonymously, using questioning in the period between March and November 2021 in Czechia. The questionnaire focused on tourism trends at selected tourism establishments in the Czechia. The questionnaire contained a total of 18 questions. In addition to the identification questions (age, gender, etc.), the questionnaire addressed questions focused on trends that customers prefer when visiting tourism facilities, which factors determine the choice of facilities, what customers perceive as innovation and whether they have encountered some innovations when visiting the selected tourism facility, etc. The research was also conducted in cooperation with The Czech Association of Hotels and Restaurants z.s. that brings together the owners and operators of hotels, boarding houses, restaurants, vocational schools, but also partners offering various products for the accommodation or restaurant operations.

The aim of this article is to identify the influence of the different factors on the repeat visits to selected international tourism enterprises based on the responses of customers in Czechia. The selected factors were divided into three main groups: service, environment, and offer, and each of these groups consists of other different factors. Primary data were analyzed using SPSS software. The research was carried out using the Survio online questionnaire system and matching the selected criteria for the research was obtained from 500 respondents. The number of correctly completed questionnaires was sufficiently representative, as there were more than 384 questionnaires according to the predefined criteria. The structure of the questionnaire was broader than the structure of the article.

Analyses of frequency responses and frequency distribution of data were used. The primary data were analyzed using SPSS software.

In this case study, the research question has been identified:

• Do professional behavior, the empathetic approach of the staff, the cleanliness and cleanliness of the company premises, the good experience and the corresponding price/quality ratio belong to significant factors that influence repeat visits to selected international tourism enterprises based on the responses of customers in Czechia?

The following hypotheses are presented:

• The decision on repeat visits depends on the gender of the respondents and the availability and location of the facility.
• The decision on repeat visits depends on the education of the respondents and the discount offers.
• Younger customers prefer music production when making repeat visits to restaurants.

The Chi-square test was used to determine whether there is a significant relationship between two nominal (categorical) variables. Based on the Chi-square test, the existence of a dependence between the quality of the service factors and the possibility of repeated purchase of the service was examined at the significance level α = 0.05. Common factors were chosen for individual tourist facilities. The hypothesis assumption was based on a comparison of critical values and statistical tests (Kajzar, 2021). H0 can be rejected at the materiality level α if the critical criterion is less than the test criterion.


χ⁢2⁢(r-1)*(c-1)⁢(1-α),

Furthermore, it is possible to use a negative value comparison (p-value) with the significance level (α) for the rejection or acceptance of Hypothesis H0.

H0 cannot be rejected if p-value > α (in our case, the significance level is 0.05). The expected value of observation eij is based on the mathematical relation (2) and (3) the value ni and the value n.j are expressed as the sum of the individual observations.


$$e_{i\,j}=\frac{n_{i}\to n_{j}}{n}$$

$$\begin{array}{cc} n_{i.}=\sum_{j=1}^{s}n_{i\,j} & n_{.j}=\sum_{i=1}^{r}n_{i\,j} \end{array}$$

## Results of the Survey

**FIGURE 1:**
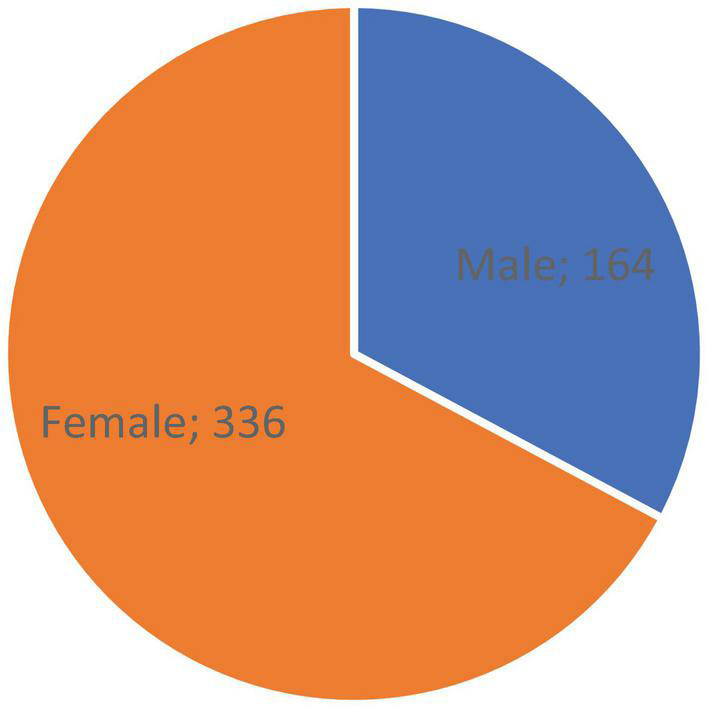
Gender. *Source*: Own processing.

The first question was dedicated to the gender of the respondents. In the survey, 500 respondents were attended, of which 164 were men and 336 were women; see Figure 1.

**FIGURE 2:**
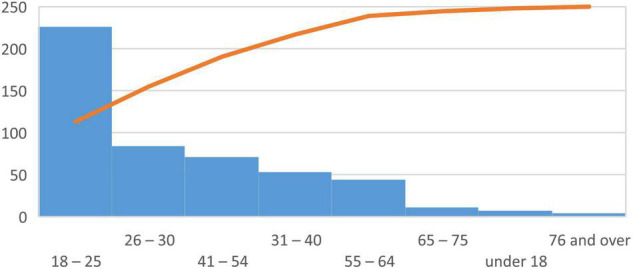
Age. *Source*: Own processing.

226 respondents were between 18 and 25 years old, 84 respondents were between 26 and 30 years old, 71 respondents were between 41 and 54 years old, and only 4 respondents were 76 years old and more; see Figure 2.

**FIGURE 3:**
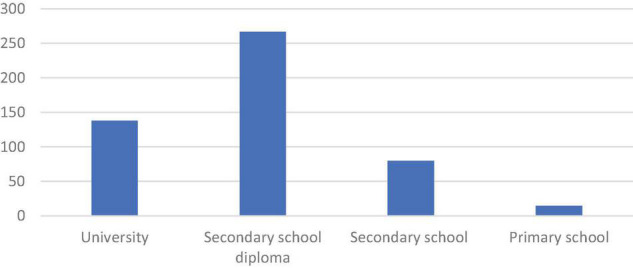
Education. *Source*: Own processing.

Another demographic question in this survey was about education. Of 500 respondents, 267 respondents had secondary school diploma, only secondary school had 80 respondents, and 138 respondents on the other hand, had university education; see Figure 3 below.

**FIGURE 4:**
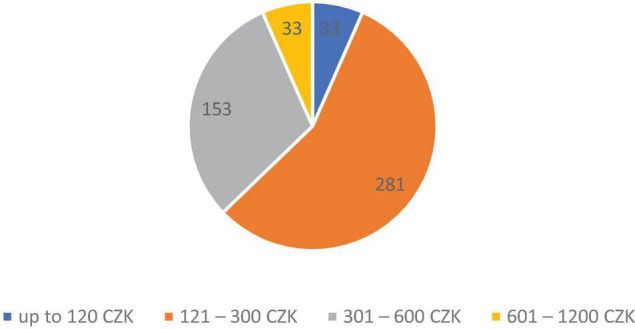
Average amount per person spent during one visit to a restaurant. *Source*: Own processing.

The survey also included a question that looked at the average amount per person spent by respondents during a visit to a restaurant. 281 respondents spent on average 121 – 300 CZK during one visit to a restaurant, 153 respondents 301 – 600 CZK, and only 33 respondents spent up to 120 CZK and also 601 – 1200 CZK; see Figure 4 below.

According to the survey in the first group (service), the following factors are extremely important to consumers: professional behavior, the empathetic approach of the staff, and the knowledge and ability to provide as much information as possible. These factors influence the highest number of repeat visits. In the second group dedicated to the environment, it belongs to the most preferred factors influencing repeat visits, for example, cleanliness and tidiness, overall atmosphere of the establishment, lighting and thermal comfort, air cleanliness, and plenty of space.

The last group is dedicated to the issue of the offer factor. Significant factors influencing repeat visits to selected international tourism enterprises as, for example, taste of food, quality of food and ingredients, corresponding ratio of price/quality, good experience, you can see below, Table 1.

**TABLE 1:** Significant factors influencing repeat visits to selected international tourism enterprises based on the responses of customers in Czechia.

Factor:	Extremely important	Not very important	Not important	I have no opinion
**Service:**	**−**	**−**	**−**	**−**
Professional behavior	384	106	6	4
Language skills	104	298	79	19
Staff empathetic approach	373	102	17	8
Offer knowledge and the ability to provide as much information as possible	373	105	12	10
**Environment:**	**−**	**−**	**−**	**−**
Facility availability and location	250	207	36	7
Parking	204	218	66	12
Attractive interior design	242	221	33	4
Cleanliness and tidiness	456	23	5	16
Plenty of space	314	173	9	4
Music production	98	263	122	17
Overall atmosphere of the establishment	383	94	20	3
The fashionability of the establishment visited	76	212	182	30
Lighting and thermal comfort, air cleanliness	367	108	16	9
**Offer:**	**−**	**−**	**−**	**−**
Attractive novelties in offer	103	300	92	5
Seasonal offer	167	273	56	4
Taste of food	446	31	19	4
Quality of food and ingredients	443	30	11	16
The way the food is served	273	198	19	10
A wide range of food and beverages	253	207	36	4
Home cooking	220	207	59	14
A range of world cuisine	143	259	73	25
Regional specialties, dishes	199	236	55	10
Specific dishes (rational diet)	139	210	120	31
Meals for children	146	158	161	35
Wide range of drinks	193	225	63	19
Discount offers	142	203	143	12
Lunch menu	244	187	67	2
Traditional and permanent menu	238	198	49	15
Games and entertainment	41	161	252	46
Promotion of the offer on the Internet	169	193	119	19
Recommendations from friends	267	195	35	3
Good experience	418	66	14	2
Attractive price level	335	131	25	9
Corresponding ratio of price/quality	443	44	5	8

*Source: Own processing.*

## Statistical Evaluation of Hypotheses

The following hypotheses are put forward:

### Hypotheses Verification 1

In this part of the article, the author deal with the evaluation of selected hypotheses.

Differences in responses obtained in the survey were checked by using the Chi square test.

Subsequently, hypotheses were established:

• Hypothesis H0: Responses according to the gender of the respondents on the availability and location of the facility do not differ.
• Alternative hypothesis H1: Responses according to the gender of the respondents on the availability and location of the facility differ.

The results of Pearson Chi-square are illustrated in the Table 2, below.

**TABLE 2:** Summarizes the result of the Chi-square test values (respondents gender availability and location of the facility).

	Value	df	Asymp. Sig. (2-sided)
Pearson Chi-Square	6,736^a^	6	0.346
Likelihood Ratio	6,812	6	0.339
N of Valid Cases	500		

*^a^6 cells (50,0%) have expected a count less than 5. The minimum expected count is 0.01.*

*Source: Own processing.*

The value of the test statistic is 6,736.

The corresponding p-value of the test statistic is *p* = 0.346.

Since the p-value is greater than our chosen significance level (α = 0.05), H0 cannot be rejected. There is not enough evidence to suggest an association between the gender of the respondents and the location of the facility.

Men and women consider the location of a restaurant equally important to their choice. Zheng (2011) found that the location of the restaurant has a positive effect on the choice of the consumer, because not every restaurant is suitable for every location. The author also agrees with Hanaysha (2016) that the convenient location is a very important factor that can influence consumers’ preferences in selecting a restaurant. The location of the restaurant depends on the type of cuisine and the style of the restaurant, and each restaurant must first define the demographic and psychographic factors of the customers.

### Hypotheses Verification 2

• Hypothesis H0: The responses according to the education respondents and the discount offers may not matter.
• Alternative hypothesis H1: The responses based on the education respondents and the discount offers may matter.

The results of Pearson Chi-square are illustrated in the Table 3, below.

**TABLE 3:** Summarizes the result of the Chi-square test values Chi-Square Tests (respondents education and discount offers).

	Value	df	Asymp. Sig. (2-sided)
Pearson Chi-Square	23,135a	9	0.006
Likelihood Ratio	23,016	9	0.006
N of Valid Cases	500		

*^a^5 cells (31,3%) have expected count less than 5. The minimum expected count is 0.36.*

*Source: Own processing.*

The value of the test statistic is 23,135.

The corresponding p-value of the test statistic is p = 0.006.

Since the p-value is not greater than our chosen significance level (α = 0.05), H0 can be rejected. There is enough evidence to suggest an association between the education of the respondents and the discount offers. On the basis of the results, we can state the following. Respondent education plays an important role in repeat visits to restaurants that offer discounts.

High school students respond to restaurant discount offers more than university students. This is largely due to the level of average wages. Even the youngest university graduates earn on average 34% more after graduation than an employee with a high school diploma. The wage gap then widens with increasing age, as salaries of employees with higher education grow more dynamically. This is also related to the amount spent in a restaurant, with university students also willing to spend a higher average amount per person spent during one visit to a restaurant, up to CZK 1,200.

### Hypotheses Verification 3

• Hypothesis H0: Younger customers do not prefer music production when making repeat visits to restaurants.
• Alternative hypothesis H1: Younger customers prefer music production when repeating visits to restaurants.

The results of Pearson Chi-square are illustrated in the Table 4, below.

**TABLE 4:** Summarizes the result of the Chi-square test values (respondents age and music production).

	Value	df	Asymp. Sig. (2-sided)
Pearson Chi-Square	39,397^a^	24	0.025
Likelihood Ratio	35,154	24	0.066
N of Valid Cases	500		

*^a^19 cells (52,8%) have expected a count less than 5. The minimum expected count is 0.07.*

*Source: Own processing.*

The value of the test statistic is 39,397.

The corresponding p-value of the test statistic is *p* = 0.025.

Since the p-value is not greater than our chosen significance level (α = 0.05), H0 can be rejected. There is enough evidence to suggest an association between the age of the respondents and the production of music. Based on the results, we can state the following. For younger customers, on repeat visits to the restaurant, music plays an more important role than for older customers.

Business owners take care to select quality music and music streaming because not only younger customers don’t just go to restaurants for the food, but for the overall experience. There is research on music around the world that advises business owners on, for example, how to make a customer feel relaxed in a bar or restaurant and how to put them in a great mood to spend. Few business operators know that they can make more money by making people feel better and more relaxed and that the whole team will benefit from a relaxed atmosphere with the charm of a smile.

## Discussion

The calling card of a good restaurant is above all its regular clientele. However, today’s businesses are increasingly demanding more stringent requirements because, according to many gastronomy experts, people have become more choosy, prefer quality over quantity, and expect a comprehensive experience when visiting a restaurant. The quality of the restaurant is also reflected in the level of service, where the waiting time for an order, free drinking water, the ability of the staff to advise on the selection of wines, their speed and responsiveness, the possibility of booking a table via the website and now more than ever, safety and health.

Based on the survey, it has been confirmed that professional behavior, the empathy of the staff, the cleanliness and tidiness of the company premises, the good experience, and the corresponding price/quality ratio belong to significant factors influencing repeat visits to selected international tourism enterprises and confirm previous studies. Individual factors that can influence a customer’s repeat visit include not only their expectations, but also, for example, the company’s image or the perceived quality of the product offered or service, quality of the human resources, and last but not least the restaurant offer.

Many tourism businesses, not only catering, are currently addressing the issue of COVID-19. As customers worry about contracting COVID-19, more are also having food taken away or delivered to their homes. Buying food on the Internet is becoming more and more common. The advantage of ordering take-away food online for customers is safety and healthy, minimal contact because the COVID-19 pandemic has elevated that preference to a priority, prices are accurate, and there is less room for error when it is time to pay the bill, and increasing customer loyalty can be done, for example, through discounts for online orders or quantity discounts.

Options, how it is possible to eat out safety and healthy, is EU Digital COVID Certificate that helped reopen events, services, travel, etc. The General Digital COVID Certificate is a certificate that can prove that the person has been vaccinated, has had COVID-19 or has a negative AG or PCR test result. Currently in the Czech Republic, only a vaccination certificate or a certificate of having had COVID-19 disease no older than 180 days can be presented. However, restaurateurs did not have to control this obligation. Restaurants were also the only establishments in Czechia where guests could present a self-test for coronavirus. The all measure, which forced businesses to check people’s infection-free status and prevented unvaccinated people from using certain services - including visiting restaurants – was in Czechia repealed on 10 February 2022. Each EU country has its own rules, so when using services in different destinations, you need to keep track of the current entry conditions.

Based on the research, three hypotheses were proposed. The first hypothesis was accepted. The decision on repeat visits depends on the gender of the respondents and the facility availability and location do not differ. Customers consider the location of a restaurant equally important to their choice, and the location of the restaurant has a positive effect on their choice because not every restaurant is suitable for all locations. The second hypothesis was rejected. Respondent education plays an important role in repeat visits to restaurants that offer discounts. High school students respond to restaurant discount offers more than university students. This is largely due to the level of average wages. Even the youngest university graduates earn on average 34% more after graduation than an employee with a high school diploma. The last third hypothesis was also rejected. For younger customers, on repeat visits to the restaurant, music plays an more important role than for older customers. Business owners take care to select quality music and music streaming because not only younger customers don’t just go to restaurants for the food, but for the overall experience. Music strongly influences buying behavior and even how much customers are willing to pay. The volume of ambient music in a restaurant also influences whether diners choose to eat light and healthy food or heavy and unhealthy food. These are all activities that must be considered by international tourism companies.

## Conclusion

The aim of this paper was to identify the influence of the different factors on the repeat visits to selected international tourism enterprises based on the responses of customers in Czechia.

In the research, the main factors were divided into three main groups including service, environment, and offer, and each of these groups consisted of other selected factors. The primary data was also analyzed using SPSS software, and three statistical hypotheses were formed, reflecting the difference relationship between the surveyed difference in responses from the customers and selected factors that may affect the repeated visits to selected international tourism enterprises in Czechia.

According to the research in the first group (service) is very significant for customers factors such as professional behavior, staff empathetic approach, offer knowledge and the ability to provide as much information as possible. These factors affect the most the repeat visits to selected international tourism enterprises in Czechia. For a tourism business to become successful, its employees should first of all be able to listen, successfully observe customer reactions, and ask the right questions. In the second group (environment), it was found that the most favorable factors influencing repeat visits to selected international tourism enterprises in Czechia are cleanliness and tidiness, overall atmosphere of the establishment, lighting and thermal comfort, air cleanliness, and plenty of space. The basis of the success of tourism businesses is the perfect level of service: delicious food and drinks prepared from quality ingredients, served by helpful and smiling staff, in a clean and pleasant environment. Therefore, businesses must pay as much attention to the environment as they do to the quality of the services they provide. The last group factor is connecting with the offer. The most preferred factors for customers in Czechia are the taste of the food, the quality of food and ingredients, corresponding ratio of price/quality, good experience, and attractive price level.

Research results consistent with previous findings, for example, of the author (Manrai and Manrai, 2009; Novotný and Duspiva, 2014; Kajzar, 2021). The factors that influence consumer buying behavior are very important not only for marketing policy because on the basis of these factors, it is possible to better overall corporate policy can be better targeted, irrespective of whether the business is in the tourism sector or outside it. The findings show that marketing and management strategies can act as support and facilitating tools to attract more potential consumers not only in the pandemic era. Restaurants have opened and many of them are trying to solve the dilemma of how to get diners to observe the mandatory distances in a clever yet creative way, so that the restaurant space is as safe as possible for them.

The most important limitations and barriers to the research were the lack of the motivation of respondents. At the time of pandemic COVID-19 to complete any questionnaire, then pandemic COVID-19 also extended the length of the research by several months and the scope of the discussions because this article is compromised in many levels compared to the work of experienced international scholars.

In further research, the author wants to focus on selected trends in tourism both from the perspective of customers and from the perspective of companies and, last but not least, on the innovations in offers that tourism businesses introduced during the COVID-19 pandemic in Central Europe.

## Data Availability Statement

The raw data supporting the conclusions of this article will be made available by the authors, without undue reservation.

## Ethics Statement

The survey was conducted in accordance with the ethical principles of scientific and research work. Ethical review and approval was not required for the study on human participants in accordance with the local legislation and institutional requirements. Written informed consent for participation was not required for this study in accordance with the national legislation and the institutional requirements.

## Author Contributions

PK performed the statistical analysis, wrote all of the manuscript, and contributed to manuscript revision, read, and approved the submitted version.

## Conflict of Interest

The author declares that the research was conducted in the absence of any commercial or financial relationships that could be construed as a potential conflict of interest.

## Publisher’s Note

All claims expressed in this article are solely those of the authors and do not necessarily represent those of their affiliated organizations, or those of the publisher, the editors and the reviewers. Any product that may be evaluated in this article, or claim that may be made by its manufacturer, is not guaranteed or endorsed by the publisher.
